# Reducing the Burden of Migraine: Safety and Efficacy of CGRP Pathway-Targeted Preventive Treatments

**DOI:** 10.3390/jcm11154359

**Published:** 2022-07-27

**Authors:** George R. Nissan, Richard Kim, Joshua M. Cohen, Michael J. Seminerio, Lynda J. Krasenbaum, Karen Carr, Vincent Martin

**Affiliations:** 1 North Texas Institute of Neurology & Headache, Plano, TX 75024, USA; 2 Premier Health Clinical Neuroscience Institute and Department of Internal Medicine and Neurology, Wright State University Boonshoft School of Medicine, Dayton, OH 45409, USA; rjkim01@gmail.com; 3 Teva Branded Pharmaceutical Products R&D, Inc., West Chester, PA 19380, USA; joshcohenmd@gmail.com (J.M.C.); lynda.krasenbaum@tevapharm.com (L.J.K.); 4 Teva Pharmaceuticals USA, Inc., Parsippany, NJ 07054, USA; michael.seminerio@tevapharm.com (M.J.S.); karen.carr03@tevapharm.com (K.C.); 5 Department of Internal Medicine, College of Medicine, University of Cincinnati, Cincinnati, OH 45267, USA; martinvt@ucmail.uc.edu

**Keywords:** migraine, CGRP, efficacy, safety and tolerability, cardiovascular safety

## Abstract

Migraine is a highly disabling and often chronic neurological disease that affects more than one billion people globally. Preventive migraine treatment is recommended for individuals who have frequent and/or disabling attacks; however, many of the medications used for migraine prevention (e.g., antiepileptics, antidepressants, antihypertensives) were not specifically developed for migraine, and often have limited efficacy or poor tolerability. Four monoclonal antibodies targeting the calcitonin gene-related peptide (CGRP) pathway, which is believed to play a crucial role in the pathophysiology of migraine, have been approved by the US Food and Drug Administration for the preventive treatment of migraine in adults. All four migraine-specific treatments have demonstrated efficacy based on reductions in monthly days with migraine for patients with both episodic and chronic migraine, including those with comorbidities. They have also demonstrated favorable safety and tolerability profiles. Based on these accounts, CGRP pathway-targeted monoclonal antibodies have the potential to revolutionize preventive treatment for patients with migraine.

## 1. Introduction

Migraine is a complex neurological disease, affecting more than one billion people worldwide, and is the second leading cause of years lived with disability overall and the leading cause of years lived with disability in young women [1]. Characterized by attacks of pulsating headache that may be exacerbated by physical activity and are accompanied by symptoms, such as photophobia, phonophobia, nausea, and vomiting [2], migraine imposes a substantial burden on activities of daily living. Migraine can be classified based on the frequency of attacks as episodic migraine (EM), with headaches occurring on less than 15 days/month, or chronic migraine (CM), with headaches occurring on 15 or more days/month for more than 3 months, and 8 of those 15 days/month fulfilling the criteria for migraine [3,4].

As well as the impact of headache and associated symptoms, migraine is associated with an increased risk of various comorbidities, which also impact patients’ well-being. For example, in the United States, migraine is associated with three-fold higher risks of depression and anxiety, along with increased risks of many other psychiatric, cardiovascular, pain, and general medical conditions, compared with the population without migraine [5]. Another burden associated with migraine is overuse of acute medications to treat migraine attacks, which can result in further escalation of headache frequency, deteriorating response to treatment, and even greater disability and quality of life (QoL) impairment [6]. 

**Figure 1:**
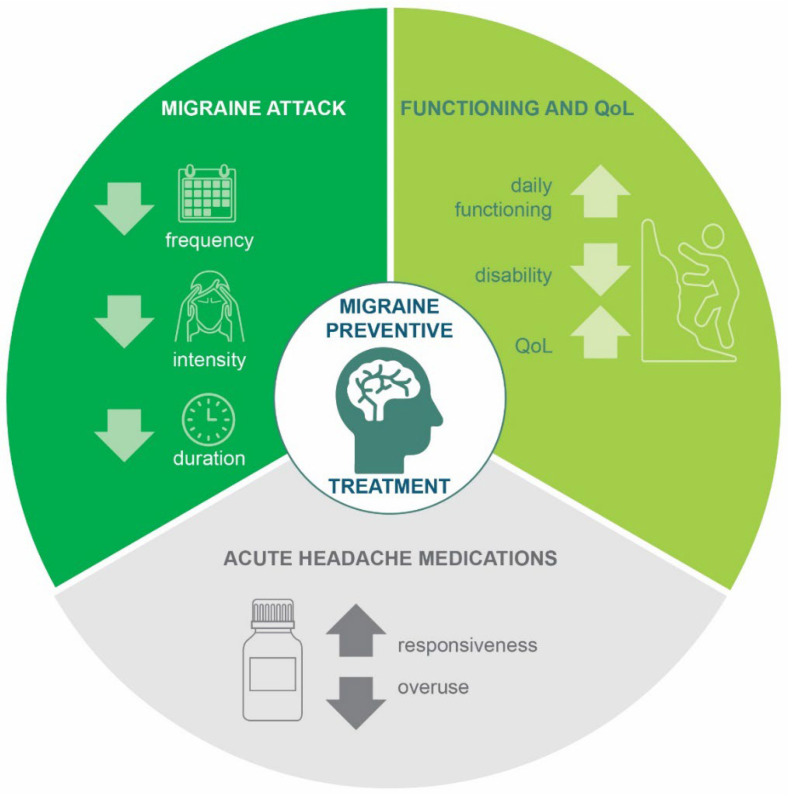
Goals of migraine preventive treatment [2]. QoL, quality of life.

Preventive migraine treatment should be considered for patients with 4 or more migraine days/month, severe migraine-related impairment, or hemiplegic migraine [2,7]. The goals of preventive treatment are to reduce attack frequency, intensity, and duration; improve responsiveness to and prevent overuse of acute headache medications; and improve daily functioning and overall QoL (Figure 1) [2]. For many years, a variety of drug classes have been used for migraine prevention, such as antiepileptics (e.g., valproate, topiramate), tricyclic antidepressants (e.g., amitriptyline, nortriptyline), serotonin norepinephrine reuptake inhibitors (e.g., venlafaxine), beta blockers (e.g., propranolol, metoprolol), calcium channel antagonists (e.g., flunarizine), serotonin antagonists (e.g., pizotifen, methysergide), and onabotulinumtoxinA [2,8]. However, none of these medications were developed specifically for migraine prevention, and they do not directly target the underlying pathophysiology of migraine. Use of these preventives requires careful management, with consideration of coexisting medical conditions and treatments, in order to avoid over- or under-treatment and possible drug-to-drug interactions. Certain medications, such as valproate or topiramate, should be avoided in women who are pregnant or trying to conceive [2]. Adherence rates to oral preventive treatments are very low, with only 26% to 29% remaining adherent at 6 months [9]. Persistence is also poor; three of every four patients discontinue oral preventive medications by 6 months [10]. The most common reasons for discontinuation of preventive treatments are a lack of efficacy (approximately 37–48% of patients discontinuing treatment) and poor tolerability (approximately 34–53% of patients discontinuing treatment) [11].

The US National Health and Wellness Survey found that despite taking acute and/or preventive migraine medications, individuals who reported 4 or more headache days/month experienced activity impairment, loss in work productivity, and increased healthcare resource use [12]. The same study demonstrated that each incremental increase in headache-free days was associated with improved function and well-being, reduction in number of days of work and home activities missed due to migraine, and reduction in indirect costs from absenteeism (missed time from work) and presenteeism (reduced productivity while at work) [13]. Thus, effective migraine preventive treatment that provides a reduction in migraine days, without a substantial burden from side effects or safety concerns, has the potential to improve many aspects of QoL for individuals with migraine.

In this article, we provide a brief introduction to targeting the calcitonin gene-related peptide (CGRP) pathway as a therapeutic approach in migraine preventive therapy, followed by an update of clinical data for CGRP pathway targeted monoclonal antibody therapies for migraine prevention, including studies published up to April 2021.

## 2. Targeting the Calcitonin Gene-Related Peptide (CGRP) Pathway

CGRP has attracted increasing attention in migraine research due to its actions as a potent vasodilator and neurotransmitter in the trigeminovascular system, activation of which is believed to underlie the generation of migraine pain and associated symptoms [14]. Four monoclonal antibodies have been developed that target the CGRP pathway. Three of these monoclonal antibodies, fremanezumab, galcanezumab, and eptinezumab, target the CGRP ligand. The fourth antibody, erenumab, targets the CGRP receptor [15]. All four are currently approved for the preventive treatment of migraine in adults. Erenumab, fremanezumab, and galcanezumab are administered by subcutaneous injection and were approved by the US Food and Drug Administration (FDA) in 2018, while eptinezumab is administered by intravenous infusion and was FDA approved in 2020 [16,17,18,19]. The pivotal double-blind, placebo-controlled trials for all four monoclonal antibodies targeting the CGRP pathway have demonstrated efficacy in migraine prevention for both EM and CM (Table 1 and Table S1) [20,21,22,23,24,25,26,27,28,29]. The pivotal trials were all of similar design, being 3- to 12-month double-blind, placebo-controlled studies enrolling patients with either EM or CM and assessing change from baseline in monthly migraine days (MMDs) as their primary endpoint. The studies did vary in the range of secondary and exploratory endpoints reported and in statistical analysis methods, which are beyond the scope of this article to describe.

In addition, the orally administered small molecules rimegepant and atogepant, which, similar to erenumab, target the CGRP receptor and block CGRP from binding, are currently being evaluated for the preventive treatment of migraine [30,31]. Rimegepant had previously been FDA approved for the acute treatment of migraine [32]. The efficacy of rimegepant and atogepant as migraine preventive treatment for patients with EM or CM (for rimegepant) and EM (for atogepant) has been demonstrated in randomized, double-blind, placebo-controlled, phase 2/3 studies [30,31]. Recently, following the results of these studies, rimegepant has been FDA approved for the preventive treatment of EM, making it the first medication approved for both the acute treatment of migraine and the prevention of migraine [33].

The addition of CGRP pathway-targeting treatments to the preventive therapeutic armamentarium for migraine may improve patient outcomes by allowing patients and clinicians a selection of different dosing options for their migraine, potentially improving adherence to and persistence with treatment, which have generally been low for daily oral preventive treatments [34]. In a survey-based study assessing patient dosing preference for migraine prevention, patients reported that they were more likely to fill their prescription and to be adherent to treatment if their preferred dosing option (either quarterly or monthly injections in this survey) was available [35]. In a separate survey following long-term treatment with fremanezumab, a higher proportion of patients (69%) reported that they preferred the lower frequency dosing schedule (quarterly) over more frequent dosing (monthly) [36].

Here, we provide an introduction to the efficacy of medications targeting the CGRP pathway followed by a review of their available safety data in migraine prevention.

## 3. Efficacy of CGRP Pathway-Targeted Treatments in Migraine Prevention

### 3.1. Reduction in Migraine Headache Days in Short-Term Studies

All 3- and 6-month studies showed a greater reduction from baseline in the mean number of MMDs and a greater proportion of patients with a 50% or greater reduction from baseline in MMDs or monthly headache days of at least moderate severity (MHDs; ≥50% response) with active treatment compared with placebo.

In patients with EM in the STRIVE and ARISE trials, erenumab provided significant reductions in MMDs compared with placebo, and up to half of patients receiving erenumab had a ≥50% response for reduction in average MMDs compared with approximately 30% of patients receiving placebo over up to 6 months (*p* < 0.01) [22,24]. Likewise, in a phase 2 trial in CM, erenumab treatment was associated with a significantly greater mean reduction in MMDs and higher ≥50% response rate compared with placebo (all *p* < 0.001; Table 1) [29]. 

In patients with EM in the HALO EM study, mean reductions in MMDs over 12 weeks were significantly higher with quarterly and monthly fremanezumab compared with placebo, and 44% to 48% of patients had a ≥50% reduction in MMDs from baseline with fremanezumab compared with 28% with placebo (all *p* < 0.001) [23]. Similarly, in patients with CM in the HALO CM study, quarterly and monthly fremanezumab provided significant reductions in MHDs compared with placebo over 12 weeks, as well as higher ≥50% response rates for the reduction from baseline in MHDs (all *p* < 0.001; Table 1) [26].

In the EVOLVE-1 and -2 trials in EM, mean reductions in MMDs were significantly greater with galcanezumab compared with placebo, and ≥50% response for the reduction in MMDs from baseline was reported in approximately 60% of galcanezumab-treated patients compared with approximately 35% to 40% of patients in the placebo groups (all *p* < 0.001) [27,28]. Likewise, in the REGAIN trial in CM, galcanezumab 120 mg and 240 mg provided significantly greater reductions and higher ≥50% response rates compared with the placebo group (all *p* < 0.001; Table S1) [21].

For patients with EM in the PROMISE-1 study, reductions in MMDs were significantly greater with eptinezumab 100 mg and 300 mg versus placebo, and up to 56% of patients showed a ≥50% response for the reduction in MMDs from baseline with eptinezumab compared with 37% with placebo (all *p* < 0.05) [20]. Similarly, in the PROMISE-2 trial of eptinezumab in CM, mean reductions in MMDs and ≥50% response rates were significantly greater with both doses of eptinezumab compared with placebo (all *p* < 0.001; Table S1) [25].

A randomized, double-blind, placebo-controlled phase 2/3 trial evaluated rimegepant 75 mg dosed every other day as a migraine preventive treatment (rimegepant, n = 373; placebo, n = 374). In that study, rimegepant treatment resulted in a mean reduction in MMDs of −4.3 compared with −3.5 with placebo (*p* = 0.0099). The proportion of patients achieving a ≥50% reduction in MMDs was also significantly greater with rimegepant (49%) compared with placebo (41%; *p* = 0.044) [31]. In a randomized, double-blind, placebo-controlled, phase 2/3 study of atogepant (10, 30, or 60 mg once daily or 30 or 60 mg twice daily), all dosing regimens for atogepant resulted in significantly greater reductions in mean MMDs, ranging from −3.6 to −4.2, compared with placebo (−2.9; all *p* ≤ 0.039). With both twice-daily atogepant dosing regimens, significantly higher proportions of patients achieved a ≥50% MMD response (30 mg twice daily, 58%; 60 mg twice daily, 62%) compared with placebo (40%; both *p* ≤ 0.034) [30].

### 3.2. Reduction in Migraine Headache Days in Long-Term Studies

Longer-term efficacy results have been published from studies for fremanezumab, galcanezumab, and eptinezumab for up to 1 year of treatment [37,38,39], and for erenumab for up to 5 years of treatment [24,40,41]. For all monoclonal antibodies targeting the CGRP pathway, efficacy seen during 12 weeks of double-blind treatment was maintained during longer term treatment. An analysis of data from the fremanezumab long-term study in patients with CM or EM confirmed that there was no evidence of a “wearing-off” effect toward the end of dosing intervals with either quarterly or monthly dosing; there was no increase in the mean number of weekly migraine days between the first 2 weeks and last 2 weeks of quarterly or monthly dosing intervals throughout a total 15 months of treatment [42]. Similarly, an analysis of data from the 6-month EVOLVE-1 and -2 studies of galcanezumab in patients with EM and the 3-month REGAIN study of galcanezumab in patients with CM, showed no evidence of “wearing off” toward the end of the monthly dosing interval; reductions in migraine days during the first 2 weeks were comparable to those during the last 2 weeks of each month of treatment [43].

### 3.3. Reduction in Disability and Improvements in QoL

The pivotal studies varied in the patient-reported outcomes that were assessed. In general, each of the CGRP monoclonal antibodies demonstrated improvement in one or more measures of headache-related disability or QoL (Table 1).

The ARISE study assessed the proportion of patients with a ≥5-point reduction in monthly average Migraine Physical Function Impact Diary (MPFID) Physical Impairment (PI) and Impact on Everyday Activities (EA) domain scores and found no significant differences between the erenumab 70 mg and placebo groups at month 3. However, other patient-reported outcomes that were used as exploratory endpoints in this study did show significant improvements with erenumab 70 mg compared with placebo at month 3, including the Headache Impact Test (HIT-6; *p* < 0.001), Modified Migraine Disability Assessment (mMIDAS; *p* = 0.021), and Migraine-Specific Quality-of-Life Questionnaire (MSQ) emotional function (*p* = 0.002), role function preventive (*p* = 0.005), and role-function restrictive (*p <* 0.001) [22]. The STRIVE study subsequently used a transformed version of MPFID-PI and -EA scores (converted to a scale of 0–100, with higher values indicating greater interference of migraine) and showed significant improvement with erenumab 70 mg and 140 mg compared with placebo (all *p* < 0.001) [24].

The HALO-EM study assessed Migraine Disability Assessment (MIDAS) scores and demonstrated a significant reduction in scores from baseline after 12 weeks of treatment with fremanezumab monthly (*p* < 0.001) and quarterly (*p* = 0.002) compared with placebo [23]. The HALO-CM study assessed HIT-6 scores and showed significant reductions with both fremanezumab regimens compared with placebo (both *p* < 0.001) [26].

The EVOLVE-1 and -2 studies in EM used MIDAS, Migraine-Specific Quality-of-Life Questionnaire role function-restrictive (MSQ-RFR), and Patient Global Impression of Severity (PGI-S) scores to assess the impact of migraine and showed significant improvements in all scores after 12 weeks of treatment with galcanezumab 120 mg and 240 mg compared with placebo (*p* ≤ 0.008) [27,28]. In the REGAIN study in CM, significant improvements were demonstrated with galcanezumab 240 mg for MSQ-RFR and PGI-S scores (both *p* < 0.0001 vs. placebo) [21].

In the PROMISE-2 study in EM, the proportion of patients with HIT-6 scores in the “severe” range (score ≥ 60) was reduced from 90% in the eptinezumab 100 mg group and 89% in the eptinezumab 300 mg group at study baseline to 51% and 43%, respectively, after 12 weeks of treatment compared with 60% in placebo group. Mean HIT-6 scores were significantly reduced compared with placebo in both 100- and 300-mg dose groups (*p* = 0.001 and *p* < 0.0001, respectively) [25].

### 3.4. Efficacy in Patients with Inadequate Response to Previous Preventive Treatments

Erenumab, fremanezumab, and galcanezumab have been investigated in 12-week placebo-controlled studies specifically in populations of patients with migraine who had experienced inadequate response (lack of efficacy or poor tolerability) to prior preventive medications or medication classes [44,45,46]. The LIBERTY study assessed erenumab in 246 patients with EM who had been treated unsuccessfully with two to four preventive treatments [44], the FOCUS trial investigated fremanezumab in 838 patients with CM or EM who had documented inadequate response to two to four separate pharmacological classes of migraine preventive medications [45], and the CONQUER study evaluated treatment with galcanezumab in 462 patients with CM or EM with failure of two to four preventive medication classes [46]. In all studies, active treatment was superior to placebo in reducing the number of MMDs and in the proportion of patients with ≥50% response, demonstrating the utility of these CGRP pathway-targeting monoclonal antibodies in patients with difficult-to-treat migraine.

### 3.5. Efficacy in Patients with Comorbidities

The three monoclonal antibodies targeting the CGRP ligand and the one monoclonal antibody targeting the CGRP receptor have shown evidence of effectiveness in subgroups of patients with migraine and comorbid conditions, including overuse of acute headache medications [47,48,49,50,51,52,53,54], moderate to severe depression [55,56,57,58,59,60], and pain disorders [61].

Erenumab treatment provided a reduction in migraine frequency and use of acute migraine-specific medication in a subgroup with medication overuse, according to the International Headache Society (IHS) criteria, in the phase 2 trial in CM (Table S2) [47]. An ongoing phase 4 randomized, placebo-controlled study is investigating the efficacy and safety of erenumab in patients with medication overuse headache, with results expected in 2022 (NCT03971071). Similarly, treatment with fremanezumab was associated with a significant reduction in MMDs in a subgroup with medication overuse in the HALO CM study (Table S2) [48]. During the 12-week treatment period, 55% and 61% of patients in the fremanezumab quarterly and monthly groups, respectively, reverted from medication overuse to no medication overuse, compared with 46% of the placebo group (*p* = 0.0389 and 0.0024, respectively). Similar results were seen in the subgroup with medication overuse in the FOCUS study, which included patients with inadequate response to two to four prior migraine preventive classes (Table S2) [49]. A pooled analysis of the galcanezumab phase 3 studies showed that in patients with baseline medication overuse, treatment with galcanezumab 120 mg or 240 mg significantly reduced MMDs and days of acute medication use (all *p* < 0.001 vs. placebo; Table S2) [50]. Likewise, in the PROMISE-2 study of eptinezumab in patients with CM, the subgroup overusing acute headache medications showed a greater reduction in MMDs with eptinezumab compared with placebo over 12 and 24 weeks of treatment [51]. In that study, 27% of patients receiving placebo, 51% receiving eptinezumab 100 mg, and 50% receiving eptinezumab 300 mg were using acute headache medications at levels below the threshold for acute medication overuse throughout 24 weeks of treatment.

In a subgroup of patients with comorbid moderate to severe depression and CM in the HALO long-term study, fremanezumab demonstrated efficacy over 52 weeks of treatment, with sustained reduction in MMDs and depression symptoms (assessed by the 9-item Patient Health Questionnaire) [58]. In the FOCUS study, significant reductions in MMDs compared with placebo, as well as improvements in depression symptoms, were observed in both EM and CM patients with comorbid moderate to severe depression (Table S2) [57,60].

In a pooled analysis of data from the galcanezumab phase 3 studies that included patients with a medical history of comorbid anxiety and/or depression, both doses of galcanezumab provided significant reductions compared with placebo in MMDs (both *p* < 0.001) for patients with EM and comorbid anxiety/depression [59]. In patients with CM and comorbid anxiety/depression, significant improvement in MMDs was seen only with the higher (240 mg) dose (Table S2). The efficacy of galcanezumab was also investigated in a subgroup of 197 patients with migraine and one or more concomitant pain disorders in the 12-week CONQUER study; the most common concomitant pain disorders were back pain, osteoarthritis, and neck pain [61]. At month 3, patients treated with galcanezumab had significantly greater reductions in MMDs (*p* < 0.01) and improvements in health status and functional migraine-specific QoL scores (*p* < 0.001) compared with placebo.

## 4. Tolerability and Safety of CGRP Pathway-Targeted Treatments in Migraine Prevention

### 4.1. Tolerability and Safety in Short-Term Studies

Across all the 3- and 6-month trials of monoclonal antibodies targeting the CGRP pathway, the overall incidence of adverse events (AEs), serious AEs (SAEs), and discontinuations due to AEs was similar in the active treatment and placebo groups (Table S3).

The most common AEs reported by erenumab-treated patients in clinical trials included injection-site pain, upper respiratory tract infection, nausea, nasopharyngitis, constipation, muscle spasms, influenza, and migraine [22,24,29,44]. In the fremanezumab trials, the most frequently reported AEs were injection-site pain, induration, and erythema [23,26,45]. With galcanezumab, injection-site reactions and nasopharyngitis were the most common AEs reported in clinical trials [21,27,28,46,62]. The most frequently reported AEs with eptinezumab treatment in clinical trials were upper respiratory tract infection, nasopharyngitis, and sinusitis [20,25]. In a 24-week analysis of eptinezumab in CM from the PROMISE-2 study, the percentages of patients experiencing any AE in the second quarterly dosing interval were lower than in the first quarterly dosing interval across all treatment groups [63]. 

In the phase 2/3 study of rimegepant for migraine preventive treatment, similar proportions of patients in the rimegepant and placebo groups, respectively, reported any AE (36% vs. 36%), an SAE (1% vs. 1%), and an AE leading to discontinuation (2% vs. 1%). The most commonly reported AEs with rimegepant treatment were nasopharyngitis, nausea, urinary tract infection, and upper respiratory tract infection [31]. In the phase 2/3 study of atogepant for migraine preventive treatment, similar proportions of patients in the atogepant groups and placebo group, respectively, reported any AE (58–66% vs. 50%), SAEs (0–1% vs. 1%), and discontinuations due to AEs (3–8% vs. 3%). The most common AEs with atogepant treatment were nausea, upper respiratory tract infection, nasopharyngitis, constipation, urinary tract infection, fatigue, and increased blood creatine phosphokinase [30].

### 4.2. Tolerability and Safety in Long-Term Studies

Longer-term safety and tolerability results have been published from studies of erenumab for up to 3 years of treatment and fremanezumab, galcanezumab, and eptinezumab for up to 1 year of treatment [37,38,39,64,65]. The favorable tolerability profiles seen in the short-term studies, with frequencies of AEs similar to the placebo arms, were maintained during longer-term open-label treatment (Table S4).

A pooled analysis of long-term data from four erenumab studies involving 2375 patients with CM or EM showed that the incidence of AEs was consistent across studies, and no new safety signals were identified during more than 3 years of treatment [65]. During long-term treatment, the individual AEs reported were generally similar to those observed during the 12-week double-blind treatment phases, but the most common events, such as injection-site reactions, constipation, and muscle spasm, were reported at lower rates during longer-term treatment. For example, injection-site reactions were reported at a rate of 17.1 per 100 patient-years during short-term erenumab treatment, compared with 5.2 per 100 patient-years during the long-term extension studies. Discontinuations due to AEs also decreased, from 5.7 events per 100 patient-years during 12 weeks of double-blind treatment to 2.4 events per 100 patient-years during long-term treatment.

In the fremanezumab long-term study, which included 1890 patients with CM or EM, injection-site reactions, such as induration, pain, and erythema, continued to be the most common AEs, reported by about one-third of patients during the 1-year treatment period [38]. Cardiovascular AEs were rare, with the most common being hypertension in 2% of patients (most of which occurred in patients with a history of high blood pressure and were not considered treatment related). AEs resulted in treatment discontinuation for 4% of patients. Around half of patients surveyed at the end of the long-term study expressed a preference for fremanezumab over previously used preventive medications due to fewer side effects, particularly in comparison with antiepileptics and tricyclic antidepressants [36].

During 1 year of treatment with galcanezumab, the most frequently reported AEs were injection-site pain and nasopharyngitis, similar to the tolerability profile seen with 12 weeks of treatment [39]. This open-label study enrolled 270 patients with migraine, most of whom (79%) had EM. AEs resulted in treatment discontinuation for 5% of patients. Patients expressed satisfaction with the impact of side effects with galcanezumab compared with prior treatments; the proportion who reported “much less side effects” increased from 42% after the first month of treatment to 58% after 1 year [66].

Over 1 year of treatment with eptinezumab, treatment-emergent AE rates were comparable to those with placebo over the full year of treatment [20,37]. The most common AEs were upper respiratory tract infection and fatigue. In the eptinezumab 100 mg and 300 mg groups, respectively, 3% and 2% of patients experienced AEs leading to discontinuation [20]. With 1 year of treatment with eptinezumab, no serious tolerability signals were identified.

### 4.3. Cardiovascular Safety

Given the presence of CGRP receptors in the cardiovascular system and the role of CGRP in regulating blood flow, the cardiovascular safety of medications targeting the CGRP pathway is of particular interest [67]. In addition, acute migraine-specific medications that are commonly used in combination with preventive treatments, such as triptans and ergots, have vasoconstrictive effects that may contribute to a higher risk of cardiovascular events [68,69].

In the pooled analysis of long-term data from four erenumab studies, the incidence of vascular AEs was similar across active treatment and placebo groups, regardless of vascular risk factors at baseline or use of acute migraine medications [70]. Across the studies, more than 70% of patients had at least one vascular risk factor, the most common being high cholesterol and obesity, and 66% were using acute migraine-specific medications. The incidence of vascular AEs during erenumab treatment was low and similar to that with placebo during double-blind treatment (Table S5). Four AEs that were adjudicated as cardiovascular in origin during open-label treatment were considered not related to erenumab treatment but to other possible underlying causes.

Fremanezumab has also been shown to be associated with a low rate of cardiovascular AEs regardless of patients’ level of cardiovascular risk or use of cardiovascular medications before starting treatment or concomitant triptan use [71,72,73]. Data were pooled from three 12-week fremanezumab studies that included 2842 patients with CM or EM and patients with prior preventive treatment failure, 18% of whom had two or more cardiovascular or cerebrovascular risk factors and 10% of whom were receiving cardiovascular medications at study baseline, and 40% of whom used triptans during study treatment. Among those patients who had two or more cardiovascular risk factors, the incidence of cardiovascular AEs was low and showed no relationship with the number of additional risk factors (Table S5) [72]. Cardiovascular tolerability in patients using triptans was similar to that in patients not using triptans [73], and in patients using cardiovascular medications, tolerability was similar across fremanezumab and placebo treatment groups [71].

A pooled analysis of data from three galcanezumab studies that included 2886 patients with CM or EM also demonstrated a similarly low incidence of cardiovascular AEs across active treatment and placebo groups (Table S5) [74]. In this analysis, 18% of the patient population were deemed at cardiovascular risk at baseline due to pre-existing conditions, most commonly hypertension or high cholesterol. The incidences of cardiovascular AEs across treatment groups were similar regardless of cardiovascular disease risk and triptan use.

The safety of monoclonal antibodies targeting the CGRP pathway has not been established in patients with known coronary or cerebrovascular disease because the proportion of patients with those disorders was quite low in clinical studies.

### 4.4. Immunogenicity

The incidence of anti-drug antibodies was low across studies of CGRP pathway-targeted monoclonal antibodies, and no relationships between the occurrence of anti-drug antibodies and treatment outcomes was reported. In the erenumab CM study, there were 14 incidences of binding antibodies in the erenumab groups (6% in the 70 mg group and 2% in the 140 mg group), while in the two EM studies, respectively, 4% to 8% and 3% of patients developed anti-erenumab antibodies [22,24,29]. In the fremanezumab pivotal trials, anti-drug antibodies were detected in two patients (<1%) with CM receiving fremanezumab (quarterly dosing) in the HALO CM trial and four patients (<1%) with EM receiving fremanezumab (monthly dosing) in the HALO EM trial [23,26,75]. In the galcanezumab CM trial, anti-drug antibodies occurred in approximately 3% of patients in both the galcanezumab 120-mg and galcanezumab 240-mg groups, respectively [21]. In the EM studies, approximately 3% to 11% of galcanezumab-treated patients developed anti-drug antibodies [27,28]. With eptinezumab 100 mg or 300 mg, anti-drug antibodies were detected in 16% to 18% of patients (Table 2) [20,25].

In the 3-year interim safety update of erenumab, 38 of 400 patients (10%) receiving erenumab 70 mg or 140 mg developed non-neutralizing antibodies [64]. In the 1-year open label extension trial of fremanezumab in CM or EM, 43 (2%) of patients developed anti-drug antibodies [38]. In the 1-year open label extension trial of galcanezumab, 16 patients (12%) in the 120 mg dose group developed anti-drug antibodies [39]. With eptinezumab 100 mg and 300 mg, respectively, 19% and 18% of patients developed anti-drug antibodies after 24 weeks of treatment (maximum), and this incidence declined to 7% and 4% after 56 weeks of treatment (Table 2) [37].

### 4.5. Tolerability and Safety in Real-World Clinical Practice

A growing number of publications are reporting real-world outcomes from those using CGRP pathway–targeted monoclonal antibodies in clinical practice, adding to the clinical trial data by including broader populations of patients with migraine. The largest proportion of patients across these studies was treated with erenumab, as this was the first medication in this class to become available in most countries. Most patients in these real-world studies had tried previous preventive medications and many had psychiatric and other comorbidities, including overuse of acute headache medication.

In a retrospective study at the University of Buffalo headache clinic involving 77 patients with CM treated with erenumab, fremanezumab, or galcanezumab and followed for up to 6 months, 63% of patients reported an AE with the most commonly reported being constipation (highest with erenumab in 16/51 patients [31%]) and injection-site reaction (highest with galcanezumab in 4/23 patients [17%]) [76]. Constipation was given as the reason for discontinuation of treatment by four patients, all of whom were using erenumab. In that study, 36 (48%) patients had a history of medication overuse headache, 44 (57%) a history of anxiety, and 32 (42%) a history of depression.

In an analysis of spontaneously reported AEs during the first 6 months post-approval of erenumab, fremanezumab, and galcanezumab from the FDA AE Reporting System (FAERS), commonly reported AEs for all three monoclonal antibodies included migraine (reporting rate per 1000 exposed patients: 4.89, 1.01, and 2.99, respectively), headache (3.32, 1.27, and 3.07, respectively), drug ineffective (3.68, 1.14, and 1.69, respectively), and injection-site pain (2.94, 0.81, and 4.90, respectively). Constipation (reporting rate: 4.90) had the second highest reporting rate for erenumab but was not within the top 10 highest reporting rates for fremanezumab or galcanezumab. Cardiovascular AEs were not among the top 10 highest reporting rates for any of the three evaluated preventive treatments [77].

Two studies from US headache clinics have reported on the experience of patients using erenumab. In a retrospective study at the John Graham Headache Center of Brigham and Women’s Hospital involving 241 patients using erenumab, 70% reported experiencing at least one side effect and 12% discontinued treatment due to side effects [78]. The most frequently reported AEs included constipation (43%), injection-site reaction (24%), and dizziness (11%). However, most patients reported they experienced more benefits than problems with treatment, and 63% planned to continue treatment. Similar results were reported from a retrospective study at the Mayo Clinic Arizona involving 101 patients treated with erenumab and followed for up to 6 months, in which 71% reported AEs, most commonly new or worsened constipation (24%) or injection-site pain (8%) [79]. While 27% of patients reported irritable bowel syndrome (IBS) at baseline, there was no evidence of an association between history of IBS and development of constipation as an AE.

Several studies on the real-world use of erenumab from headache clinics in Italy, Germany, and the United Kingdom have been recently reported [80,81,82,83,84,85,86,87,88]. Tolerability findings were generally consistent across these European sites, with around one-third of erenumab-treated patients reporting AEs. Across all studies, the most frequently reported AE was constipation, by 10% to 24% of patients. Injection-site reactions were also reported, typically by fewer than 10% of patients. Severe AEs leading to discontinuation were reported in only one of these studies, in which 19 of 162 patients (12%) discontinued erenumab due to severe AEs, nine with severe constipation, five with consistent headache worsening after the injection, two with flu-like symptoms, and one each with whole body itchiness, mood deterioration, and new-onset hypertension [80].

In general, the burden of AEs with erenumab in real-world studies appeared higher than that reported in the phase 2/3 clinical studies. In particular, constipation was reported for 1% to 3% of patients randomized to erenumab in the phase 2 and 3 clinical trials [16,22,24,29,44] and at a rate of <2 per 100 patient-years in the pooled analysis of long-term extension studies [65], compared with up to 40% of patients in the studies from headache clinics. The product label for erenumab currently includes a statement of safety concern for “constipation with serious complications” [16].

The ongoing prospective GARLIT study is currently evaluating the use of galcanezumab in patients treated at six Italian headache clinics [89]. Of 66 patients to complete 3 months of treatment, minor adverse effects, such as itching and dizziness, were reported by 9.8% during the first month, 7.3% in the second month, and 2.4% in the third month. No SAEs or events requiring treatment discontinuation were reported.

Additional real-world experience with the three monoclonal antibodies targeting CGRP (fremanezumab, galcanezumab, and eptinezumab) and the monoclonal antibody targeting the CGRP receptor (erenumab), will continue to provide valuable information on the tolerability and safety of these agents in the long-term preventive treatment of migraine.

## 5. Future Directions

Continuing to gather real-world data will be helpful in further establishing the long-term efficacy and safety of the CGRP monoclonal antibodies, determining how these medications compare to other preventive therapies from patients’ perspective, and ascertaining the potential utility of combination therapy with other treatments.

For example, recent retrospective data from 257 patients with CM treated at a US headache clinic suggested benefits from adding a CGRP monoclonal antibody therapy to onabotulinumtoxinA treatment. Average monthly headache frequency was reduced by 9.3 days following at least 2 cycles of onabotulinumtoxinA, and then a further 3.5 to 4.0 days over 6 to 12 months after the addition of a CGRP monoclonal antibody, along with accompanying clinically meaningful improvements in migraine-related disability; no new safety concerns were identified [90]. The evidence from this study supports similar findings from previous chart reviews in smaller cohorts of patients that suggested benefits from combination therapy, including reduced headache frequency, disability, and acute headache medication use [91,92,93]. While these studies looked at the effects of adding a CGRP monoclonal antibody to an existing onabotulinumtoxinA regimen, it would also be of interest to investigate the effectiveness of adding onabotulinumtoxinA therapy to CGRP monoclonal antibody treatment compared with switching therapies.

Ultimately, a proportion of patients with inadequate responses to previously available preventive therapies may also experience unsatisfactory changes in headache frequency and disability with CGRP monoclonal antibodies. For these patients, the development of new treatments targeting alternative pathways is eagerly awaited [94]. Pituitary adenylate cyclase-activating polypeptide (PACAP) is a neuropeptide expressed in the trigeminovascular system. Infusion of PACAP has been shown to cause migraine-like headache in individuals with migraine, prompting development of a monoclonal antibody to the PACAP type 1 (PAC1) receptor, AMG301 [95]. However, treatment with AMG301 proved to be no more effective than placebo in reducing headache frequency in a phase 2 study involving patients with inadequate response or poor tolerability to previous preventive treatments [96]. Meanwhile, monoclonal antibodies to the PACAP38 ligand, ALD1910 (NCT04197349) and LY3451838 (NCT04498910) are currently in clinical development, with no study results available [97,98]. The development of agents targeting the orexinergic system, nitric oxide signaling pathway, and metabotropic glutamate receptor has unfortunately stalled due to either lack of efficacy or safety concerns in phase 2 studies [94,99].

As new migraine preventive treatments do not appear to be imminently on the horizon, further prospective studies may be warranted to explore alternative treatment options for patients who experience inadequate efficacy with CGRP-targeted therapies and other currently available preventives.

## 6. Conclusions

Monoclonal antibodies targeting the CGRP pathway have the potential to revolutionize migraine preventive treatment with targeted efficacy in reducing the frequency, severity, and duration of migraine attacks, while maintaining favorable tolerability and safety profiles. Further observational and prospective studies are needed to elucidate which patients are most likely to obtain benefit from these agents as well as alternative strategies for patients whose migraine attacks show unsatisfactory response to these treatments.

## Figures and Tables

**Table 1:** Summary of the CGRP pathway-targeting monoclonal antibody short-term pivotal trials efficacy results.

CGRP Pathway-Targeting mAbStudy Name/Description	MMDs	≥50% Reduction in MMDs	Disability and QoL Measures
**Erenumab**			
STRIVE phase 3 study in EM [24] ARISE phase 3 study in EM [22] Phase 2 study in CM [29]	Significantly greater reduction from baseline in MMDs with erenumab vs. PBO in all studies (all *p* < 0.001)	Demonstrated in 40–50% of erenumab-treated patients vs. ≤30% of PBO-treated patients (all *p* < 0.001)	No significant difference between erenumab and PBO in proportion of patients with ≥5-point reduction in MPFID-EA and MPFID-PI scores [22] Significant improvement in transformed MPFID-EA and MPFID-PI scores vs. PBO (all *p* < 0.001) [24] Significant reductions in HIT-6, mMIDAS, MSQ-EF, MSQ-RPF, MSQ-RFR scores (exploratory endpoints) [22]
**Fremanezumab**			
HALO EM phase 3 study [23] HALO CM phase 3 study [26]	Significantly greater reduction from baseline in MMDs with fremanezumab vs. PBO in all studies (all *p* < 0.001)	Demonstrated in 38–48% of fremanezumab-treated patients vs. ≤28% of PBO-treated patients (all *p* < 0.001)	Significant reductions in MIDAS scores with fremanezumab vs. PBO in EM (*p* ≤ 0.002) [23] Significant reductions in HIT-6 scores with fremanezumab vs. PBO in CM (*p* < 0.001) [26]
**Galcanezumab**			
EVOLVE-1 phase 3 study in EM [28] EVOLVE-2 phase 3 study in EM [27] REGAIN phase 3 study in CM [21]	Significantly greater reduction from baseline in MMDs with galcanezumab vs. PBO in all studies (all *p* < 0.001)	Demonstrated in 28–62% of galcanezumab-treated patients vs. ≤15–39% and 15% of PBO-treated patients (all *p* < 0.001)	Significant reductions in MIDAS, MSQ-RFR, and PGI-S scores in EM (*p* ≤ 0.008) [27,28] Significant reductions in MSQ-RFR and PGI-S scores in CM (*p* ≤ 0.006) [21]
**Eptinezumab**			
PROMISE-1 phase 3 study in EM [20] PROMISE-2 phase 3 study in CM [25]	Significantly greater reduction from baseline in MMDs with eptinezumab vs. PBO in all studies (all *p* < 0.05)	Demonstrated in 50–61% of eptinezumab-treated patients vs. ≤39% of PBO-treated patients (all *p* < 0.05)	Signficant improvement in HIT-6 scores (*p* < 0.001) [25]

CGRP, calcitonin gene-related peptide; CM, chronic migraine; EM, episodic migraine; HIT, Headache Impact Test; MIDAS, Migraine Disability Assessment; mMIDAS, Modified Migraine Disability Assessment; MMD, monthly migraine day; MPFID-EA, Migraine Physical Function Impact Diary Impact on Everyday Activities; MPFID-PI, Migraine Physical Function Impact Diary Physical Impairment; MSQ-EF, Migraine-Specific Quality-of-Life Questionnaire emotional function; MSQ-RFP, Migraine-Specific Quality-of-Life Questionnaire role function-preventive; MSQ-RFR, Migraine-Specific Quality-of-Life Questionnaire role function-restrictive; PBO, placebo; PGI-S, Patient Global Impression of Severity; QoL, quality of life.

**Table 2:** Incidence of anti-drug antibodies and neutralizing antibodies ^a^ across phase 2/3 trials of monoclonal antibodies targeting the CGRP pathway [20,21,22,23,24,25,26,27,28,29,37,38,39,40,41,64,75].

	3- to 6-Month Phase 2/3 Studies				≥1-Year Phase 3 Studies	
Medication	Anti-Drug Antibodies in CM	Neutralizing Antibodies in CM	Anti-Drug Antibodies in EM	Neutralizing Antibodies in EM	Anti-Drug Antibodies	Neutralizing Antibodies
Erenumab	2–6%	0%	3–8%	0–1%	6–10% ^b^	<1% ^b^
Fremanezumab	<1%	0%	<1%	<1%	2% ^b^	<1% ^b^
Galcanezumab	3%	2%	3–11%	3%	12% ^b^	12% ^b^
Eptinezumab	16–18%	6–7%	14–18%	10%	18–19% ^c^	8% ^c^

CGRP, calcitonin gene-related peptide; CM, chronic migraine; EM, episodic migraine. ^a^ The clinical significance of neutralizing antibodies is not known. ^b^ EM and CM patients. ^c^ CM patients.

## Data Availability

All data described in this manuscript is publically available.

## Supplementary Materials

The following supporting information can be downloaded at: https://www.mdpi.com/article/10.3390/jcm11154359/s1, Table S1: Efficacy results from the pivotal trials of the efficacy of CGRP pathway-targeting monoclonal antibodies for CM or EM; Table S2. Efficacy of CGRP pathway-targeting monoclonal antibodies for patients with migraine and comorbid depression or medication overuse; Table S3. Overall frequency of AEs in the short-term trials of the monoclonal antibodies targeting the CGRP pathway in the prevention of migraine; Table S4: Most frequently reported AEs during long-term treatment with erenumab, fremanezumab, and galcanezumab; Table S5: Treatment-emergent cardiovascular AEs, n (%), in pooled analyses of clinical studies of the monoclonal antibodies targeting the CGRP pathway.
