# Supplementary material for: Reducing the Burden of Migraine: Safety and Efficacy of CGRP Pathway-Targeted Preventive Treatments
Source: J Clin Med. 2022 Jul 27;11(15):4359. doi: 10.3390/jcm11154359 (PMC9369309; doi:10.3390/jcm11154359)
Supplement: Supplementary file 1 [file jcm-11-04359-s001.zip › jcm-1671710-supplementary.pdf]

Table S1. Efficacy results from the pivotal trials of the efficacy of CGRP pathway-targeting monoclonal antibodies for CM or EM.

CGRP Pathway-Targeting mAb Study Name/Description	Migraine Classification	Study Design	Dosing Schedule	Randomized Patients	Primary Endpoint	Responder Rate
**Erenumab**						
STRIVE phase 3 study [24]	EM	RDBPC 6-month study	Monthly SC	955	LSM change from BL in MMD during months 3-6:<br>• PBO, −1.8<br>• Erenumab 70 mg, −3.2<br>• Erenumab 140 mg, −3.7<br>Both $p < 0.001$ vs. PBO	≥50% reduction in MMD from BL during months 3-6:<br>• PBO, 27%<br>• Erenumab 70 mg, 43%<br>• Erenumab 140 mg, 50%<br>Both $p < 0.001$ vs. PBO
ARISE phase 3 study [22]	EM	RDBPC 12-week study	Monthly SC	577	LSM change from BL in MMD at week 12:<br>• PBO, −1.8<br>• Erenumab 70 mg, −2.9<br>$p < 0.001$ vs. PBO	≥50% reduction in MMD from BL at week 12:<br>• PBO, 30%<br>• Erenumab 70 mg, 40%<br>$p = 0.010$ vs. PBO
Phase 2 study [29]	CM	RDBPC 12-week study	Monthly SC	667	LSM change from BL in MMD during weeks 9-12:<br>• PBO, −4.2<br>• Erenumab 70 mg, −6.6<br>• Erenumab 140 mg, −6.6<br>Both $p < 0.001$ vs. PBO	≥50% reduction in MMD from BL during weeks 9-12:<br>• PBO, 23%<br>• Erenumab 70 mg, 40%<br>• Erenumab 140 mg, 41%<br>Both $p < 0.001$ vs. PBO
**Fremanezumab**						
HALO EM phase 3 study [23]	EM	RDBPC 12-week study	Month and quarterly SC	875	LSM change from BL in MMD during 12 weeks:<br>• PBO, −2.2<br>• Quarterly fremanezumab, −3.4<br>• Monthly fremanezumab, −3.7<br>Both $p < 0.001$ vs. PBO	≥50% reduction in MMD from BL during 12 weeks:<br>• PBO, 28%<br>• Quarterly fremanezumab, 44%<br>• Monthly fremanezumab, 48%<br>Both $p < 0.001$ vs. PBO
HALO CM phase 3 study [26]	CM	RDBPC 12-week study	Monthly and	1,130	LSM change from BL in MHD during 12 weeks:	≥50% reduction in MHD from BL during 12 weeks:

			quarterly SC		• PBO, −2.5<br>• Quarterly fremanezumab, −4.3<br>• Monthly fremanezumab, −4.6<br>Both $p < 0.001$ vs. PBO	• PBO, 18%<br>• Quarterly fremanezumab, 38%<br>• Monthly fremanezumab, 41%<br>Both $p < 0.001$ vs PBO
**Galcanezumab**						
EVOLVE-1 phase 3 study [28]	EM	RDBPC 6-month study	Monthly SC	862	LSM change from BL in MMD across months 1-6<br>• PBO, −2.8<br>• Galcanezumab 120 mg, −4.7<br>• Galcanezumab 240 mg, −4.6<br>Both $p < 0.001$ vs. PBO	≥50% reduction in MMD across months 1-6<br>• PBO, 39%<br>• Galcanezumab 120 mg, 62%<br>• Galcanezumab 240 mg, 61%<br>Both $p < 0.001$ vs. PBO
EVOLVE-2 phase 3 study [27]	EM	RDBPC 6-month study	Monthly SC	922	LSM change from BL in MMD across months 1-6<br>• PBO, −2.3<br>• Galcanezumab 120 mg, −4.3<br>• Galcanezumab 240 mg, −4.2<br>Both $p < 0.001$ vs. PBO	≥50% reduction in MMD across months 1-6<br>• PBO, 36%<br>• Galcanezumab 120 mg, 59%<br>• Galcanezumab 240 mg, 57%<br>Both $p < 0.001$ vs. PBO
REGAIN phase 3 study [21]	CM	RDBPC 12-week study	Monthly SC	1,117	LSM change from BL in MMD across months 1-3<br>• PBO, −2.7<br>• Galcanezumab 120 mg, −4.8<br>• Galcanezumab 240 mg, −4.6<br>Both $p < 0.001$ vs. PBO	≥50% reduction in MMD across months 1-3<br>• PBO, 15%<br>• Galcanezumab 120 mg, 28%<br>• Galcanezumab 240 mg, 28%<br>Both $p < 0.001$ vs. PBO
**Eptinezumab**						
PROMISE-1 phase 3 study [20]	EM	RDBPC 12-week study	Quarterly IV	898	Mean change from BL in MMD during weeks 1–12	≥50% reduction in MMD during weeks 1–12

					• PBO, −3.2<br>• Eptinezumab 30 mg, −4.0<br>• Eptinezumab 100 mg, −3.9<br>• Eptinezumab 300 mg, −4.3<br>All $p < 0.05$ vs. PBO	• PBO, 37%<br>• Eptinezumab 30 mg, 50%<br>• Eptinezumab 100 mg, 50%<br>• Eptinezumab 300 mg, 56%<br>All $p < 0.05$ vs. PBO
PROMISE-2 phase 3 study [25]	CM	RDBPC 12-week study	Quarterly IV	1121	Mean change from BL in MMD during weeks 1–12<br>• PBO, −5.6<br>• Eptinezumab 100 mg, −7.7<br>• Eptinezumab 300 mg, −8.2<br>Both $p < 0.001$ vs. PBO	≥50% reduction in MMD during weeks 1–12<br>• PBO, 39%<br>• Eptinezumab 100 mg, 58%<br>• Eptinezumab 300 mg, 61%<br>Both $p < 0.001$ vs. PBO

BL, baseline; CGRP, calcitonin gene-related peptide; CM, chronic migraine; EM episodic migraine; IV, intravenous; LSM, least-squares mean; mAb, monoclonal antibody; MHD, monthly headache days; MMD, monthly migraine days; PBO, placebo; RDBPC, randomized, double-blind, placebo-controlled; SC, subcutaneous.

Table S2. Efficacy of CGRP pathway-targeting mAbs for patients with migraine and comorbid depression or medication overuse.

Comorbidity CGRP Pathway-Targeting mAb	Subgroup	Study Design	Dosing Schedule	Number of Patients	Outcomes	
**Comorbid medication overuse**						
					**Change in MMD or MHD**	**Change in Days with Acute Medication Use**
Erenumab	CM with medication overuse [47][a]	RDBPC 12-week phase 3 study	Monthly SC	274	LSM change from BL in MMD at 12 weeks:<br>• PBO, −3.5<br>• Erenumab 70 mg, −6.6<br>• Erenumab 140 mg, −6.6<br>Both $p < 0.001$ vs. PBO	LSM change from BL in days of migraine-specific acute medication use:<br>• PBO, −2.1<br>• Erenumab 70 mg, −5.4<br>• Erenumab 140 mg, −4.9<br>Both $p < 0.001$ vs. PBO
Fremanezumab	CM with medication overuse [48]	RDBPC 12-week phase 3 study	Monthly or quarterly SC	587	LSM change from BL in MMD at 12 weeks:<br>• PBO, −2.8<br>• Quarterly fremanezumab, −4.8<br>• Monthly fremanezumab, −5.2<br>Both $p < 0.001$ vs. PBO	LSM change from BL in days of any acute medication use:<br>• PBO, −3.1<br>• Quarterly fremanezumab, −4.9<br>• Monthly fremanezumab, −5.5<br>Both $p < 0.001$ vs. PBO
	CM/EM with medication overuse and inadequate response to 2–4 prior migraine preventive treatment classes [49]	RDBPC 12-week phase 3 study	Monthly or quarterly SC	435	LSM change from BL in MMD during 12 weeks:<br>• PBO, −0.5<br>• Quarterly fremanezumab, −3.3<br>• Monthly fremanezumab, −4.6<br>Both $p \leq 0.001$ vs. PBO	LSM change from BL in days of any acute medication use during 12 weeks:<br>• PBO, −0.8<br>• Quarterly fremanezumab, −3.9<br>• Monthly fremanezumab, −4.9<br>Both $p < 0.001$ vs. PBO

	CM or EM with medication overuse [52-54]	RDB 12-month phase 3 extension study	Monthly or quarterly SC	CM: 602<br>EM: 100	Mean change from BL in MMD at month 12:<br>CM<br>• Quarterly fremanezumab, −7.5<br>• Monthly fremanezumab, −8.2<br>EM<br>• Quarterly fremanezumab, −5.9<br>• Monthly fremanezumab, −5.1	Mean change from BL in days of any acute medication use at month 12:<br>CM<br>• Quarterly fremanezumab, −8.1<br>• Monthly fremanezumab, −8.2<br>EM<br>• Quarterly fremanezumab, −6.1<br>• Monthly fremanezumab, −5.3
Galcanezumab	CM with medication overuse [50]	RDBPC 12-week study	Monthly SC	708	LSM change from BL in MMD over months 1–3:<br>• PBO, −2.3<br>• Galcanezumab 120 mg, −4.8<br>• Galcanezumab 240 mg, −4.5<br>Both $p < 0.001$ vs. PBO	LSM change from BL in MMD with acute medication use over months 1–3:<br>• PBO, −2.5<br>• Galcanezumab 120 mg, −5.3<br>• Galcanezumab 240 mg, −4.7<br>Both $p > 0.05$ vs. PBO
	EM with medication overuse [50]	RDBPC 6-month study	Monthly SC	334	LSM change from BL in MMD over months 1–6:<br>• PBO, −2.7<br>• Galcanezumab 120 mg, −6.3<br>• Galcanezumab 240 mg, −5.8<br>Both $p < 0.001$ vs. PBO	LSM change from BL in MMD with acute medication use over months 1–6:<br>• PBO, −2.7<br>• Galcanezumab 120 mg, −6.3<br>• Galcanezumab 240 mg, −5.9<br>Both $p < 0.001$ vs. PBO
Eptinezumab	CM with comorbid MOH [51]	RDBPC 24-week study	Quarterly IV	431	LSM change from BL in MMD over weeks 1–12:<br>• PBO, −5.4<br>• Eptinezumab 100 mg, −8.4<br>• Eptinezumab 300 mg, −8.6<br>Both $p < 0.001$ vs. PBO	—
**Comorbid depression**						

					Change in MMD or MHD	Change in anxiety or depression symptoms
Erenumab	EM and history of anxiety/depression [55]	RDBPC 6-month phase 3 study	Monthly SC	193	LSM change from BL in MMD during months 4–6:<br>• PBO, −1.3<br>• Erenumab 70 mg, −4.2<br>• Erenumab 140 mg, −4.1<br>Both $p < 0.001$ vs. PBO	—
Fremanezumab	CM and moderate to severe depression at BL (PHQ-9 ≥10) [56]	RDBPC 12-week phase 3 study	Monthly or quarterly SC	219	LSM change from BL in MHD during 12 weeks:<br>• PBO, −2.2<br>• Quarterly fremanezumab, −5.3<br>• Monthly fremanezumab, −5.5<br>Both $p < 0.001$ vs. PBO	LSM change from BL in PHQ-9 scores to Week 12:<br>• PBO, −9.2<br>• Quarterly fremanezumab, −10.9<br>• Monthly fremanezumab, −9.8<br>Both $p > 0.05$ vs. PBO
	CM/EM, moderate to severe depression at BL (PHQ-9 ≥10), and inadequate response to 2–4 prior migraine preventive treatment classes [57]	RDBPC 12-week phase 3b study	Monthly or quarterly SC	154	LSM change from BL in MMD during 12 weeks:<br>• PBO, 0.2<br>• Quarterly fremanezumab, −3.2<br>• Monthly fremanezumab, −3.9<br>Both $p < 0.01$ vs. PBO	LSM change from BL in PHQ-9 scores during weeks 9–12:<br>• PBO, −4.9<br>• Quarterly fremanezumab, −6.7<br>• Monthly fremanezumab, −7.8<br>Both $p > 0.05$ vs. PBO
	CM and moderate to severe depression at BL (PHQ-9 ≥10) [58]	RDB 12-month phase 3 extension study	Monthly or quarterly SC	231	Mean change from BL in MMD during 12 months:<br>• Quarterly fremanezumab, −7.2<br>• Monthly fremanezumab, −8.6	Mean change from BL in PHQ-9 scores at month 12:<br>• Quarterly fremanezumab, −10.3<br>• Monthly fremanezumab, −10.5
Galcanezumab	CM or EM and history of anxiety/depression [59]	RDBPC 12-week (CM) or 6-month (EM) phase 3 studies	Monthly SC	CM: 316<br>EM: 461	LSMD vs PBO for the change from BL in MMD during months 1–6:<br>CM:	—

					• Galcanezumab 120 mg, −1.5<br>• Galcanezumab 240 mg, −1.9<br>$p < 0.05$ for galcanezumab 240 mg vs. PBO<br>EM:<br>• Galcanezumab 120 mg, −2.1<br>• Galcanezumab 240 mg, −1.9<br>Both $p < 0.001$ vs. PBO	

BL, baseline; CGRP, calcitonin gene-related peptide; CM, chronic migraine; EM episodic migraine; IV, intravenous; LSM, least-squares mean; mAb, monoclonal antibody; MMD, monthly migraine days; PBO, placebo; PHQ-9, 9-item Patient Health Questionnaire; RDB, randomized, double-blind; RDBPC, randomized, double-blind, placebo-controlled; SC, subcutaneous.

Table S3. Overall frequency of AEs in the short-term trials of the monoclonal antibodies targeting the CGRP pathway in the prevention of migraine.

**Erenumab**			
Phase 2 (CM), n (%) [29]	Placebo (n = 282)	Erenumab 70 mg (n = 190)	Erenumab 140 mg (n = 188)
Any AE	110 (39)	83 (44)	88 (47)
SAE	7 (2)	6 (3)	2 (1)
Discontinuation due to AE	2 (<1)	0	2 (1%)
STRIVE (EM), n (%) [24]	Placebo (n = 319)	Erenumab 70 mg (n = 314)	Erenumab 140 mg (n = 319)
Any AE	201 (63)	180 (57)	177 (56)
SAE	7 (2)	8 (3)	6 (2)
Discontinuation due to AE	8 (3)	7 (2)	7 (2)
ARISE (EM), n (%) [22]	Placebo (n = 289)	Erenumab 70 mg (n = 283)	–
Any AE	158 (55)	136 (48)	
SAE	5 (2)	3 (1)	
Discontinuation due to AE	1 (<1)	5 (2)	
LIBERTY prior preventive failure (EM), n (%) [44]	Placebo (n = 124)	Erenumab (n = 119)	
Any AE	67 (54)	65 (55)	
SAE	1 (1)	2 (2)	
Discontinuation due to AE	1(1)	0	
**Fremanezumab**			
HALO (CM), n (%) [26]	Placebo (n = 375)	Fremanezumab quarterly (n = 376)	Fremanezumab monthly (n = 379)
Any AE	240 (64)	265 (70)	270 (71)
SAE	6 (2)	3 (<1)	5 (1)
Discontinuation due to AE	8 (2)	5 (1)	7 (2)
HALO (EM), n (%) [23]	Placebo (n = 293)	Fremanezumab quarterly (n = 291)	Fremanezumab monthly (n = 290)
Any AE	171 (58)	193 (66)	192 (66)
SAE	7 (2)	3 (1)	3 (1)
Discontinuation due to AE	5 (2)	5 (2)	5 (2)
FOCUS prior inadequate response (CM or EM), n (%) [45]	Placebo (n = 277)	Fremanezumab quarterly (n = 276)	Fremanezumab monthly (n = 285)
Any AE	134 (48)	151 (55)	129 (45)
SAE	4 (1)	2 (<1)	4 (1)
Discontinuation due to AE	3 (1)	1 (<1)	4 (1)
**Galcanezumab**			
REGAIN (CM), n (%) [21]	Placebo (n = 558)	Galcanezumab 120 mg (n = 273)	Galcanezumab 240 mg (n = 282)
Any AE	279 (50)	159 (58)	160 (57)

SAE	4 (<1)	1 (<1)	5 (2)
Discontinuation due to AE	6 (1)	1 (<1)	4 (1)
EVOLVE-1 (EM), n (%) [28]	Placebo (n = 432)	Galcanezumab 120 mg (n = 206)	Galcanezumab 240 mg (n = 220)
Any AE	261 (60)	135 (66)	149 (68)
SAE	5 (1)	6 (3)	0
Discontinuation due to AE	10 (2)	9 (4)	7 (3)
EVOLVE-2 (EM), n (%) [27]	Placebo (n = 461)	Galcanezumab 120 mg (n = 226)	Galcanezumab 240 mg (n = 228)
Any AE	287 (62)	147 (65)	163 (72)
SAE	5 (1)	5 (2)	7 (3)
Discontinuation due to AE	8 (2)	5 (2)	9 (4)
CONQUER prior preventive failure (CM or EM), n (%) [46]	Placebo (n = 230)	Galcanezumab 120 mg (n = 232)	
Any AE	122 (53)	119 (51)	
SAE	2 (1)	2 (1)	
Discontinuation due to AE	0	1 (<1)	
**Eptinezumab**			
PROMISE-2 (CM), n (%) [25]	Placebo (n = 366)	Eptinezumab 100 mg (n = 356)	Eptinezumab 300 mg (n = 350)
Any AE	171 (47)	155 (44)	182 (52)
SAE	3 (<1)	7 (1)	
Discontinuation due to AE	2 (<1)	3 (<1)	8 (2)
PROMISE-1 (EM), n (%) [20]	Placebo (n = 222)	Eptinezumab 100 mg (n = 223)	Eptinezumab 300 mg (n = 224)
Any AE	132 (60)	141 (63)	129 (58)
SAE	6 (3)	11 (2)	
Discontinuation due to AE	6 (3)	6 (3)	5 (2)

AE, adverse event; CGRP, calcitonin gene-related peptide; CM, chronic migraine; EM, episodic migraine; SAE, serious adverse event.

Table S4. Most frequently reported AEs during long-term treatment with erenumab, fremanezumab, and galcanezumab.

**Erenumab**			
Pooled long-term extension phases of 4 studies [65]	Erenumab 70 mg (n = 1891) 1782.5 pt-yr	Erenumab 140 mg (n = 933) 822.9 pt-yr	All (n = 2375) 2605.3 pt-yr
n (exposure-adjusted patient incidence rate per 100 pt-yr)			
Viral upper respiratory tract infection	264 (16.6)	119 (15.8)	351 (15.5)
Upper respiratory tract infection	151 (9.0)	61 (7.8)	192 (7.9)
Sinusitis	79 (4.6)	44 (5.5)	119 (4.8)
Arthralgia	68 (3.9)	25 (3.1)	92 (3.7)
Back pain	70 (4.1)	24 (3.0)	91 (3.6)
Urinary tract infection	67 (3.8)	28 (3.5)	90 (3.6)
Influenza	73 (4.2)	20 (2.5)	88 (3.5)
Migraine	61 (3.5)	25 (3.1)	85 (3.3)
Nausea	48 (2.7)	33 (4.1)	79 (3.1)
Fatigue	41 (2.3)	32 (4.0)	72 (2.8)
Injection-site pain	60 (3.5)	11 (1.4)	69 (2.7)
Dizziness	46 (2.6)	16 (2.0)	61 (2.4)
Bronchitis	46 (2.6)	13 (1.6)	59 (2.3)
Cough	38 (2.2)	12 (1.5)	48 (1.9)
Oropharyngeal pain	35 (2.0)	10 (1.2)	44 (1.7)
Constipation	24 (1.4)	17 (2.1)	41 (1.6)
Injection-site erythema	13 (0.7)	10 (1.2)	22 (0.9)

**Fremanezumab**				
	CM		EM	
52-week HALO long-term study [38]	Quarterly (n = 550)	Monthly (n = 558)	Quarterly (n = 394)	Monthly (n = 386)
n (%), occurring in >4% of patients in any group				
Injection-site induration	165 (30)	196 (35)	113 (29)	145 (38)
Injection-site pain	157 (29)	182 (33)	118 (30)	123 (32)
Injection-site erythema	138 (25)	171 (31)	85 (22)	103 (27)
Upper respiratory tract infection	77 (14)	72 (13)	59 (15)	45 (12)
Nasopharyngitis	64 (12)	61 (11)	41 (10)	51 (13)
Injection-site hemorrhage	42 (8)	44 (8)	17 (4)	28 (7)
Sinusitis	40 (7)	39 (7)	19 (5)	18 (5)
Urinary tract infection	39 (7)	28 (5)	22 (6)	24 (6)
Injection-site pruritis	26 (5)	39 (7)	15 (4)	35 (9)
Bronchitis	23 (4)	25 (4)	21 (5)	14 (4)
Influenza	22 (4)	30 (5)	11 (3)	11 (3)

**Galcanezumab**		
52-week long-term open-label study [39]	Galcanezumab 120 mg (n = 129)	Galcanezumab 240 mg (n = 141)
n (%), occurring in ≥5% of patients in either group		
Injection-site pain	22 (17.1)	28 (19.9)
Nasopharyngitis	23 (17.8)	18 (12.8)

Upper respiratory tract infection	9 (7.0)	21 (14.9)
Injection-site reaction	15 (11.6)	13 (9.2)
Back pain	12 (9.3)	15 (10.6)
Sinusitis	14 (10.9)	13 (9.2)
Nausea	10 (7.8)	9 (6.4)
Injection-site erythema	9 (7.0)	9 (6.4)
Arthralgia	8 (6.2)	8 (5.7)
Influenza	8 (6.2)	8 (5.7)
Dizziness	5 (3.9)	9 (6.4)
Injection-site bruising	5 (3.9)	8 (5.7)
Myalgia	8 (6.2)	3 (2.1)
Weight increased	7 (5.4)	4 (2.8)

AE, adverse event; CM, chronic migraine; EM, episodic migraine; pt-yr, patient-year.

Table S5. Treatment-emergent CV AEs, n (%), in pooled analyses of clinical studies of the monoclonal antibodies targeting the CGRP pathway.

**Erenumab: pooled analysis of 4 clinical studies [70]**			
AE category, n (%)	Placebo (n = 1043)	Erenumab 70 mg (n = 893)	Erenumab 140 mg (n = 507)
Ischemic CNS vascular conditions	0	0	1 (0.2)
Ischemic heart disease	0	0	0
Peripheral arterial disease	0	0	0
Hypertension	9 (0.9)	7 (0.8)	1 (0.2)
**Fremanezumab: pooled analysis of 3 clinical studies [72]**			
AE category, n (%)	Placebo	Quarterly fremanezumab	Monthly fremanezumab
Patients with ≥2 CV risk factors	(n = 169)	(n = 174)	(n = 156)
Cardiac disorders	3 (2)	1 (1)	1 (0.6)
Vascular disorders	1 (0.6)	3 (2)	4 (3)
Patients with ≥3 CV risk factors	(n = 61)	(n = 68)	(n = 54)
Cardiac disorders	1 (2)	0	1 (2)
Vascular disorders	1 (2)	2 (3)	2 (4)
Patients with ≥4 CV risk factors	(n = 17)	(n = 20)	(n = 18)
Cardiac disorders	0	0	0
Vascular disorders	0	0	0
**Galcanezumab: pooled analysis of 3 clinical studies [74]**			
CV AE, n (%)[a]	Placebo (n = 1451)	Galcanezumab 120 mg (n = 705)	Galcanezumab 240 mg (n = 730)
Any event	42 (2.9)	18 (2.6)	24 (3.3)
Cardiac arrhythmias	20 (1.4)	5 (0.7)	10 (1.4)
Cardiac failure	4 (0.3)	1 (0.1)	1 (0.1)
Cardiomyopathy	14 (0.1)	4 (0.6)	8 (1.1)
CNS vascular disorders	0	0	1 (0.1)
Embolic or thrombotic events	4 (0.3)	0	4 (0.6)
Hypertension	18 (1.2)	9 (1.3)	7 (1.0)
Ischemic heart disease	1 (0.1)	2 (0.3)	1 (0.1)
Pulmonary hypertension	0	2 (0.3)	1 (0.1)
Torsade de pointes/QT prolongation	8 (0.6)	2 (0.3)	3 (0.4)

AE, adverse event; CGRP, calcitonin gene-related peptide; CNS, central nervous system; CV, cardiovascular. [a]Patients with at least 1 broad or narrow preferred term.
